# TGF-β participates choroid neovascularization through Smad2/3-VEGF/TNF-α signaling in mice with Laser-induced wet age-related macular degeneration

**DOI:** 10.1038/s41598-017-10124-4

**Published:** 2017-08-29

**Authors:** Xiaolei Wang, Wei Ma, Song Han, Zhaoyang Meng, Lu Zhao, Yi Yin, Yanling Wang, Junfa Li

**Affiliations:** 1 Department of Ophthalmology, Beijing Friendship Hospital, Capital Medical University, Beijing, 100050 PR China; 2 Department of Neurobiology and Center of Stroke, Beijing Institute for Brain Disorders, Capital Medical University, Beijing, 100069 PR China; 3 Beijing Stomatological Hospital, Capital Medical University, Beijing, 100050 PR China

## Abstract

Choroidal neovascularization(CNV) is the most severe complication in Age-related macular degeneration(AMD) and the most common cause of irreversible blindness in the elderly in developed world. The aim of this study was to identify the effect of transforming growth factor-β(TGF-β) and Smad2/3-VEGF/TNF-α signaling on CNV angiopoiesis, and to explore TGF-β inhibitors on the development of CNV in a CNV mouse model. Fundus fluorescein angiography(FFA) was used to evaluate the laser-induced CNV formation. The histology of CNV lesions stained with hematoxylin-eosin(HE) was obtained. The immunofluorescent staining was performed to determine TGF-β protein expression. The expressions of TGF-β, phosphorylated Smad2/3, VEGF and TNF-α were determined by using Western blot analysis. The CNV areas were analyzed by using fluorescein stain on RPE/choroid-sclera flat mounts. We found the levels of TGF-β protein expression increasingly reached the peak till 3rd week during the CNV development. The protein levels of VEGF and TNF-α also increased significantly in CNV mice, which were inhibited by a synthetic TGF-β inhibitor LY2157299 or a natural TGF-β inhibitor Decorin. The phosphorylated Smad2/3 levels increased significantly in CNV mice, but this response was profoundly suppressed by the TGF-β inhibitors. Here we have demonstrated that TGF-β/Smad signaling plays an important role in Laser-induced CNV formation through down-regulation of VEGF and TNF-α expressions, suggesting TGF-β inhibitors may provide an alternative to traditional methods in wet AMD treatment.

## Introduction

Age-related macular degeneration (AMD) is the most common cause of irreversible blindness and an improving public health problem due to aging population in the developed world^1, 2^. Factors implicated in AMD include age, genetic predisposition, oxidative stress, diet, smoking and activation of complement^3^. Two forms of AMD are recognized as nonexudative (dry) and exudative (wet) types. Dry AMD is characterized by the presence of drusen, debris accumulated underneath the retina, pigmentary changes and geographic atrophy in some eyes (GA)^4^. In contrast, wet AMD is characterized by aberrant angiogenesis within the subretinal space, referred to as choroid neovascularization (CNV) or retinal angiomatous proliferation (RAP) within the retina, and usually causes severe and rapid vision loss^5^. CNV is the most severe complication and the hallmark of wet AMD, these vessels usually break through Bruch’s membrane to enter the subretinal space, causing retinal pigment epithelium (RPE) detachment, subretinal or intraretinal hemorrhage, fibrovascular scarring, resulting in retinal tissue damage and loss of vision^6^.

Although CNV angiopoiesis is a series of complex pathological processes and still remains unclear, experimental and clinical studies have revealed that a variety of cytokines, chemokines and endothelial adhesion molecules play crucial roles in the development of CNV^7–9^. Several angiogenic factors, such as vascular endothelial growth factor (VEGF), platelet-derived growth factor (PDGF), angiopoietin, stromal derived factor (SDF)-1, b-fibroblast growth factor (FGF), pigment epithelium-derived factor (PEDF) and thrombospondin-1 were confirmed to be key molecules in pathological angiogenesis^10–12^. The increased expression of VEGF in CNV has been identified, and the VEGF inhibition is a highly effective treatment in wet AMD. In clinical practice, wet AMD patients are currently treated with intravitreal anti-VEGF agents including Lucentis (Ranibizumab), Eylea (Aflibercept) and bevacizumab (avastin)^13, 14^. However, these treatments have various limitations such as the requirement of repeat intravitreal injections. Furthermore strategies targeting only one of multiple angiogenic factors are not sufficient to control the disease process^15, 16^. Thus, development of an alternative strategy to achieve combination therapy that blocks other signaling pathways is more likely to produce a greater therapeutic benefit for wet AMD.

Recently, there is increasing evidence that indicates that some inflammatory cytokines such as interleukin (IL)-1 and IL-6, tumor necrosis factor (TNF)-α and transforming growth factor beta (TGF-β) have an effect on CNV^17, 18^. The TGF-β superfamily comprises a large number of multifunctional polypeptides that participate in many diverse biological processes, including cell proliferation, differentiation, neuronal growth, angiogenesis, inflammation, fibrotic processes and immune surveillance^19^. TGF-β is a pleiotropic cytokine that binds to membrane receptors bearing serine/threonine kinase activity, namely TGF-β receptors type I and type II, which were located in the retinal ganglion cell layer and Müller glia in mouse retina, and signals through both Smad-dependent and Smad-independent pathways^20^. In retina, TGF-β is produced by RPE cells and pericytes^21^, and the increased intravitreal transforming growth factor beta 1 (TGF-β_1_) levels appear to be related to retinal angiogenesis^22^. While TGF-β has been shown to be able to induce angiogenesis *in vivo* ^23, 24^, the mechanisms are not well defined. It has been reported that TGF-β may significantly stimulate strong VEGF secretion in human RPE cell cultures^18, 25^. In addition, TGF-β_1_ has also been implicated in angiogenesis as a regulator of endothelial cell proliferation and macrophage infiltration as well as deposition and proteolytic remodeling of extracellular matrix (ECM)^26^, resulting in activated angiogenesis and vascular remodeling.

Considering the crucial roles of TGF-β in neovascularization, we hypothesize that TGF-β may work in concert with other cytokines in VEGF up-regulation, and selective blockade of TGF-β would abate CNV development. To test this hypothesis, the Laser photocoagulation was applied to establish the experimental CNV mouse model to mimic wet AMD. The changes of TGF-β, TNF-α and VEGF protein expressions, as well as Smad2/3 phosphorylation levels were observed in RPE-choroid complex of mice after Laser-induced CNV formation. In addition, two TGF-β specific inhibitors LY2157299 and Decorin were used to determine whether TGF-β inhibition could prevent the formation of CNV. We found that TGF-β/Smad pathway through regulation of pro-angiogenic factors played a critical role in the development of experimental CNV, suggesting TGF-β/Smad signaling pathway blockage may provide a novel, effective auxiliary treatment for AMD.

## Materials and Methods

Male C57BL/6 J mice at age of 6–8 weeks old and weighing 20–25 g were used in the study. Animals were housed in metal breeding cages with free access to food and water in a room with a 12-h/12-h light/dark cycle. The humidity and temperature were maintained at 55% and 23 °C, respectively. All the animal procedures were performed with strict adherence to the guidelines set by the Animal Care and Use Committee of Capital Medical University{SCXK(Jing) 2016-0011} and the Association for Research in Vision and Ophthalmology Statement for the Use of Animals in Ophthalmic and Vision Research. The experimental protocols were approved by Capital Medical University licensing committee. All reasonable efforts were made to minimize suffering. Except for individually indicated agents and antibodies in the text, all chemicals were purchased from Sigma-Aldrich (St. Louise, MO 63103, USA).

### Laser-induced CNV and Drug Treatment

Anesthesia was induced by intraperitoneal injection of pentobarbital (0.05 mg/g body weight), and pupils were dilated with topical 5% tropicamide (Santen, Osaka, Japan). Following mydriasis, mice were placed on a platform under the slit lamp, and the corneas were anesthetized with 0.5% proparacaine hydrochloride eye drops (Alcon Laboratories, Elkridge, MD 21075, USA). Laser photocoagulation (532 nm laser, 120 mW, 100 ms duration, 50 µm spot size) was performed bilaterally in each mouse. Laser spots were applied in a standard fashion around the optic nerve using a slit lamp delivery system (Lumenis 1000, Salt Lake City, UT 84104, USA) with a handheld cover slip as a contact lens. Photocoagulation lesions (4–6/eye) were delivered in a peripapillary distribution at a distance of 1- to 2-disc diameters from the optic nerve, avoiding major vessels. The appearance of a cavitation bubble by laser treatment, which indicates disruption of Bruch’s membrane, is an important factor in obtaining CNV; therefore, only burns in which a bubble was produced were included in subsequent experiments. Spots containing hemorrhage or failing to develop a bubble at the laser site were excluded from the analysis. The eye was subsequently coated with erythromycin eye ointment. The experimentally sham treated mice were assigned as normal (Nor) control.

To investigate the effect of TGF-β inhibitor on the formation of CNV, mice were randomly assigned to treatment and sham control groups. The TGF-β inhibitor LY2157299 (S2230; Selleck, Houston, TX 77230, USA), a new and potent specific TGF-β type I receptor inhibitor of the family of pyrazoles, was dissolved in dimethylsulfoxide (DMSO) and diluted with phosphate buffered saline (PBS, 0.07% DMSO). Right after Laser injury, LY2157299 was delivered to mice by intraperitoneal injection with the dose of 1.5, 3.0 and 6.0 μg/g body weight per day or the same volume of vehicle (0.07% DMSO) for 7 days, 14 days, 21 days and 28 days to evaluate the inhibitory effect on CNV size^27^. Based on preliminary results, 3.0 μg/g LY2157299 was used for all the experiments. This study also evaluates the effect of Decorin, a naturally occurring TGF-β inhibitor. Decorin (D-8428; Sigma-Aldrich, St. Louise, MO 63103, USA) was dissolved in PBS and diluted to reach a final dose of 10 μg/μl. After the photocoagulation, a 1 μl intravitreal injection of decorin or control PBS was administered using a microliter syringe^28^.

### Fundus Photography and Fundus Fluorescein Angiography

One week after Laser photocoagulation, fundus examinations were performed under systemic anesthesia and pupil dilation using a digital fundus camera (Heidelberg Retina Angiograph II, Heidelberg Engineering, Heidelberg, Germany), and the Laser lesions were studied using fundus fluorescein angiography (FFA) to evaluate CNV development. FFA images were taken at 2–5 min after intraperitoneal injection of 0.1 ml of 2.5% fluorescein sodium (Alcon, Houston, TX 77054-1309, USA).

### Analysis of Lesion Area in RPE/choroid Flat Mounts

One to four weeks after the CNV Laser procedure, mice were euthanized by cervical dislocation and eyes were enucleated and fixed at 4% paraformaldehyde solution for 1–2 h. Flat mounts of RPE-choroid complex were obtained by removing the anterior segments and the neural retina under an operation microscope. Then retinal and choroid tissues were washed in PBS containing 1% BSA, 0.2% Tween-20 (AppliChem GmbH, Darmstadt 64291, Germany) and 0.1% Triton-X 100. Eyecups were incubated with 0.5% fluorescein-isothiocyanate (FITC)-isolectin B4 (Vector Laboratories Inc., Burlingame, CA 94010, USA) overnight at 4 °C to label invading choroid vessels and then washed in PBS three times. After staining, the eyecups were flattened by four to six radial cuts from the edge to the equator and flat mounted with the scleral side facing down onto a microscope slide. The flatmounts were analyzed with fluorescence microscope (Olympus, Hamburg, Germany). The size of FITC-isolectin B4 positive CNV areas was quantified with Olympus CellF software. Lesions with obvious hemorrhage or bridging CNV were excluded. Images were measured and confirmed by two reviewers in a masked procedure.

### Western Blot Analysis

Antibodies used in this study are listed as follows: rabbit anti-TGF-β polyclonal antibody (1:500; catalogue number ab66043), rabbit anti-VEGF polyclonal antibody (1:1,000; catalogue number ab46154) and rabbit anti-TNF-α polyclonal antibody (1:500; catalogue number ab9635, Abcam, Cambridge, UK); rabbit anti-Smad2/3 polyclonal antibody (1:1,000; catalogue number #5678) and anti-Phospho-Smad2/3 monoclonal antibody (1:1,000; catalogue number #8828, Cell Signaling Technology, Boston, MA 02241–3843, USA); mouse anti-β-actin monoclonal antibody (1:3,000; Sigma-Aldrich Corp. St. Louis, MO 63103, USA); and the horseradish peroxidase conjugated goat anti-rabbit or anti-mouse IgG as secondary antibody (1:4,000; Stressgen Biotechnologies Corporation, Victoria BC, Canada).

Tissues harvested from the retina, RPE and choroid of control and experimental mice were homogenized and solubilized in ice-cold PBS containing 1% protease inhibitors and 1% NP-40. Protein concentration in the supernatant was quantified by BCA (Pierce, Rockford, IL 61101, USA). Equal amounts of protein were loaded for SDS-PAGE (10% SDS gel). Proteins were then electrophoresed and transferred onto polyvinylidene difluoride membrane (GE Healthcare, UK) and blocked with 10% non-fat milk in Tween-20/Tris-buffered salt solution (TTBS, 20 mM Tris–Cl, pH 7.5, 0.15 M NaCl and 0.05% Tween-20) for 1 h. Following incubation with the primary and secondary antibodies, the Enhanced Chemiluminescence kit (GE Healthcare, UK) was used to detect the signals. The amount of proteins were quantified by densitometry and normalized to β-actin as an internal standard.

### Histopathological Examination and Immunohistochemistry

After each animal was sacrificed by cervical dislocation, the eyeballs were enucleated and fixed at 4% Paraformaldehyde solution, conventionally dehydrated and embedded in paraffin wax. The optic nerve parallel to the sagittal plane at the Laser photocoagulation position was selected, and slices with a thickness of 6.0 μm were prepared continuously. The sections were stained with hematoxylin-eosin (HE), and then were observed and photographed under a light microscope.

Mouse eyes were enucleated and fixed in 4% paraformaldehyde. Frozen sections with thickness of 10-μm were blocked with 3% (wt/vol) BSA and incubated with rabbit anti-TGF-β polyclonal antibody. The fluorescent-labeled secondary antibodies were followed and imaged under fluorescence microscope (Olympus, Hamburg, Germany).

### Statistical Analyses

Data are expressed as mean ± SEM. Significant differences between groups were determined by one-way ANOVA in combination with all pair wise multiple comparison procedures using Bonferroni test. In all cases, values of p < 0.05 were considered as statistically significant.

## Results

### Increased CNV Formation in the Retina of Mice after Laser Injury

**Figure 1:**
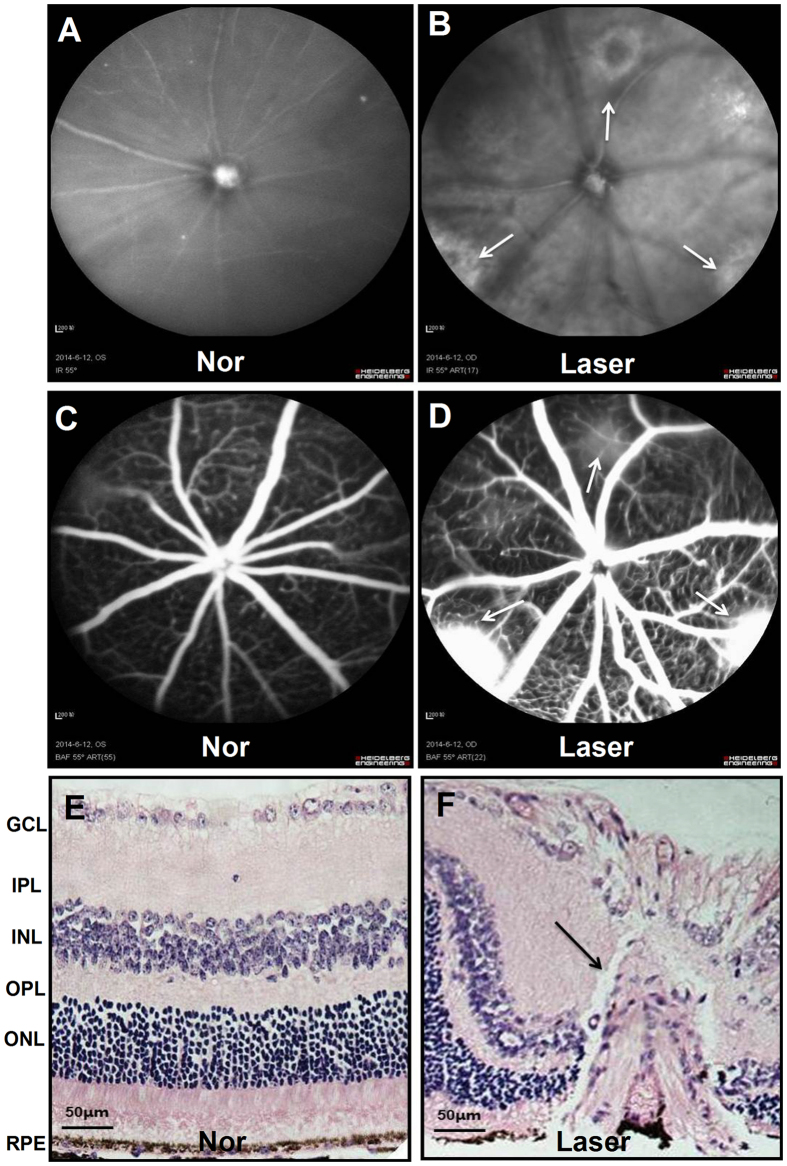
Morphological evaluation of mouse fundus at one week after Laser injury. The typical results of fundus photographs (**A**,**B**) and fundus fluorescein angiography (FFA, **C**,**D**) revealed that there were hyperfluorescence leakage in the Laser injured spots (white arrows) of fundus at one week after photocoagulation; the hematoxylin-eosin (HE) staining results (**E**,**F**, scale bar = 50 μm) showed that Laser-induced CNV presented vascular plexus with wide blood vessel lumen, which originated from the choroid and grew towards the bottom of the retina (black arrows) when compared with that of experimentally normal (Nor) control (sham treated).

One week after Laser photocoagulation, fundus photographs and fundus fluorescein angiography (FFA) were carried out in experimentally sham treated normal control (Nor) and experimental (Laser) mice to examine CNV formation and fluorescein leakage (Fig. 1A–D). The Laser spots showed a central depigmented crater surrounded by irregular hyperpigmentation in fundus photograph and evident fluorescein leakage in FFA, which indicated CNV formation at one week after Laser injury.

The histological analysis of retina stained with HE (Fig. 1E and F) also confirmed that the CNV began to form on day 7 after Laser-induced rupture of Bruch’s membrane. Damages to the RPE, outer nuclear layer, outer plexiform layer and inner nuclear layer were seen in all CNV lesions, and the newly formed fibrovascular complex, which consisted of collagen fibers, fibroblasts, endothelial cells, RPE cells and newly formed vessels, extended towards the subretinal space (Fig. 1F).

Flat mounts were used to evaluate the presence and area of clearly demarcated isolectin positive CNV from one to four weeks post-laser injury, which showed the time course of CNV formation in Table 1. The mean CNV area was 11933.20 ± 2041.07 µm^2^ at day 7, and then significantly increased at day 14 (24313.99 ± 3308.46 µm^2^), day 21 (30932.12 ± 2894.39 µm^2^) and day 28 (33075.49 ± 2585.29 µm^2^) (P < 0.05) in the retina of mice post-laser injury.

**Table 1:** Assessment of Laser-induced choroid neovascularization (CNV) formation through fluorescein-isothiocyanate (FITC)-isolectin B4 staining in RPE/choroid flat mounts of mice.

Time (d)	Case Numbers	Light spots	Area of CNV (μm^2^, x ± s)	Increased multiple of CNV area	
				Relative ratio with fixed base	Link relative ratio
7	4	48	11933.20 ± 2041.07	1.00	1.00
14	4	48	24313.99 ± 3308.46	2.04	2.04
21	4	48	30932.12 ± 2894.39	2.59	1.27
28	4	48	33075.49 ± 2585.29	2.77	1.07

### Increased TGF-β Protein Expression in the Retina Following Laser-induced CNV Formation

**Figure 2:**
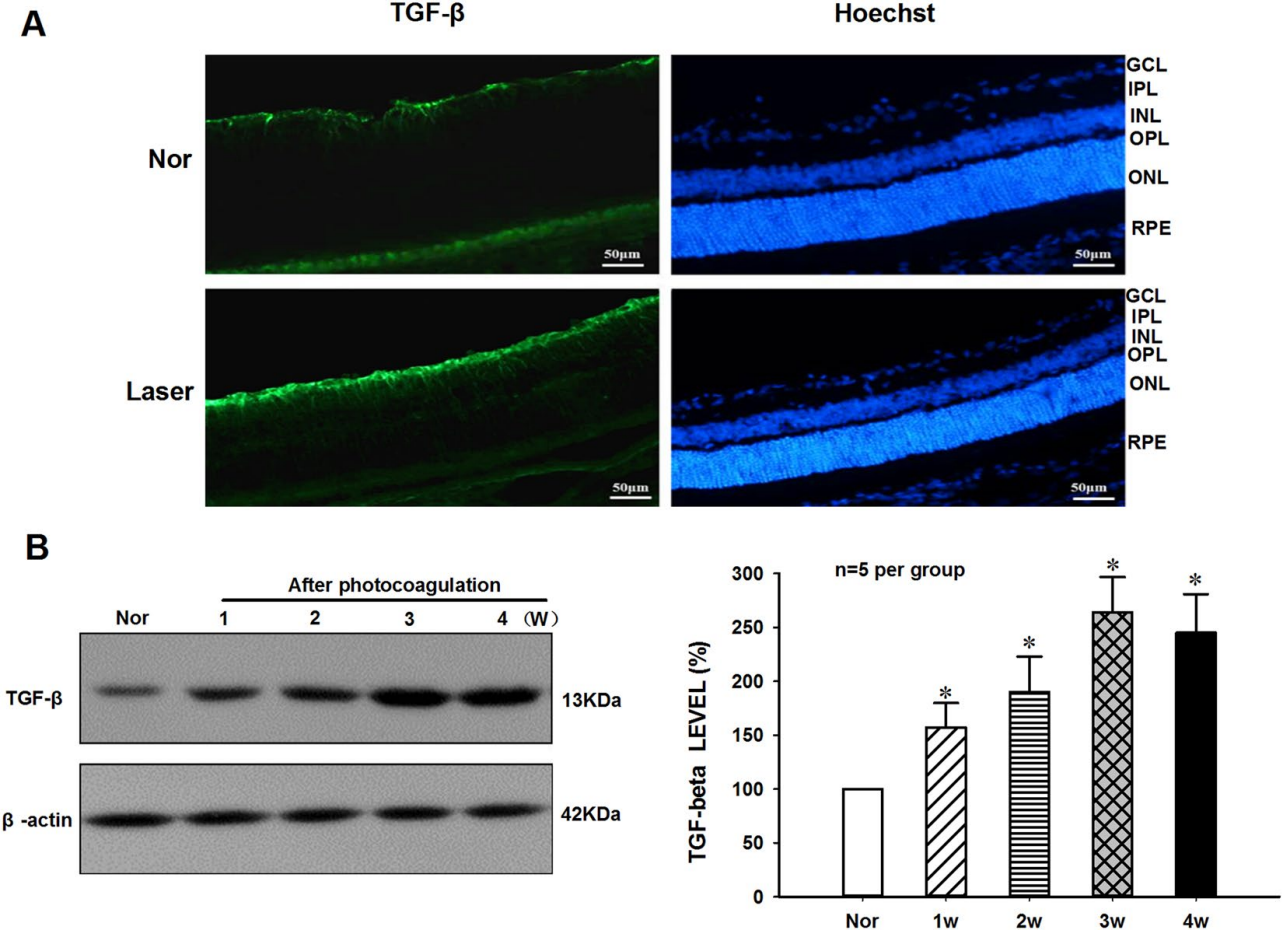
Increased expression of TGF-β in retina of mice after Laser-induced CNV formation. (**A**) Flat mounts were fluorescently labeled with TGF-β antibody (green) and the nuclear staining with Hoechst (blue). Low levels of TGF-β expression were detected in ganglion cells of retina and choroid of Nor control mice. However, the strong immunoreactivity could be observed in the ganglion cells, outer nuclear layer, inner nuclear layer of retina and choroid at two weeks after Laser injury (Scale bars = 50 μm). (**B**) Typical result of Western blot showed changes of TGF-β protein levels in RPE/choroid/sclera isolated from the mice with or without Laser-induced CNV formation. (**C**) The quantitative analysis results demonstrated that the TGF-β protein levels were significantly up regulated at one week and reached a peak at three weeks in RPE/choroid/sclera of mice after Laser photocoagulation. The β-actin was used as loading control and reference protein, and the ratio of TGF-β/β-actin in Nor mice was set as 100%. *P < 0.05 vs Nor, n = 5 per group.

As shown in Fig. 2A, the immunofluorescence staining results indicated that TGF-β staining was weakly observed in the retinal ganglion cells and choroid of normal control mice, while the TGF-β protein expression remained at high level in the ganglion cell layer, outer nuclear layer, inner nuclear layer and choroid of mice after 2-weeks photocoagulation. Furthermore, the TGF-β protein expression levels in retina-choroid complex during the development of laser-induced CNV formation was determined by using Western blot (Fig. 2B). The quantitative analysis results in Fig. 2C demonstrated that the protein expression levels of TGF-β increased significantly [F(4,20) = 28.025, p < 0.05, n = 5 per group] from 1 to 4 weeks and reached the peak at day 21 in the retina-choroid complex of mice after Laser-induced CNV formation when compared with that of normal controls.

### Effect of LY2157299 on VEGF and TNF-α Protein Expression and Smad2/3 Phosphorylation Levels in Retina Following Laser-induced CNV Formation

TGF-β protein expression level is elevated in the RPE and choroidal blood vessels of AMD patients and may promote the occurrence of angiogenesis by regulating the activity of vascular endothelial cells and the expression of VEGF^18^. To elucidate whether TGF-β affects angiogenic and inflammatory molecules related to the CNV formation, the effect of TGF-β type I receptor-specific inhibitor LY2157299 on VEGF and TNF-α protein expression and Smad2/3 phosphorylation levels were determined in retina during CNV formation.

**Figure 3:**
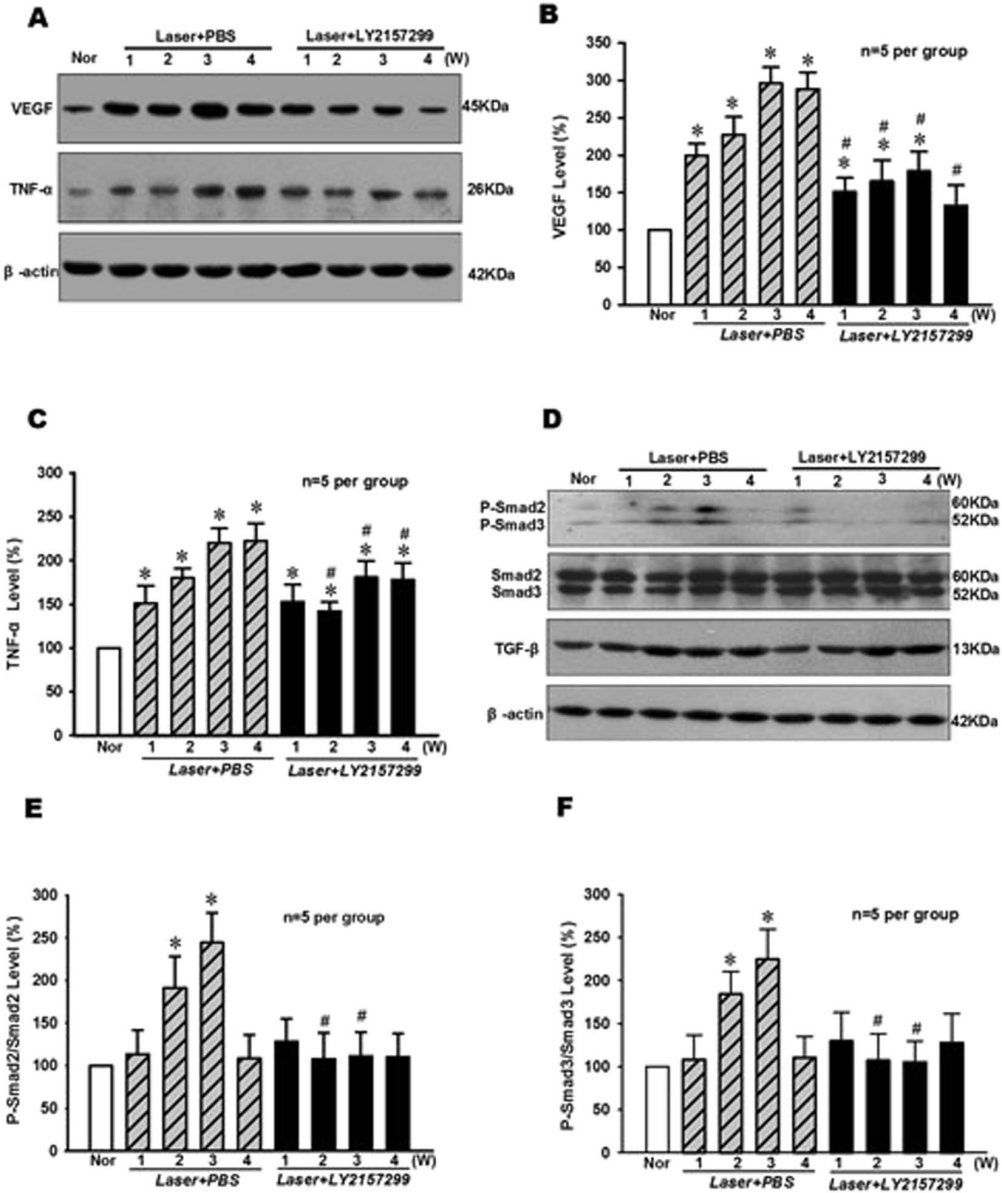
Effect of TGF-β inhibitor LY2157299 on the levels of VEGF and TNF-α protein expression and Smad2/3 phosphorylation in RPE-choroid complex of mice after Laser-induced CNV formation. (**A**) The typical result of Western blot showed changes of VEGF and TNF-α protein expressions. The quantitative analysis results demonstrated that the protein expression levels of VEGF (**B**) and TNF-α (**C**) were significantly elevated and peaked at 3–4 weeks following CNV induction, but TGF-β chemical inhibitor LY2157299 (intraperitoneal injection, 3.0 μg/g BW) treatment could significantly suppress the increase of VEGF and TNF-α protein expressions in RPE-choroid complex of mice at 1–4 weeks after Laser-induced CNV formation. (**D**) The representative result of Western blot showed the effect of LY2157299 on Smad2/3 phosphorylation and TGF-β protein expression levels in RPE-choroid complex of Laser-treated mice. The quantitative analysis results indicated that LY2157299 could significantly inhibit the increase of p-Smad2/Smad2 (**E**) and p-Smad3/Smad3 (**F**) levels in RPE-choroid complex of mice at 2–3 weeks after Laser-induced CNV formation. *P < 0.05 vs Nor; ^#^P < 0.05 vs corresponding PBS treatment; n = 5 per group.

As shown in Fig. 3A–C, the protein expression levels of growth factor VEGF and pro-inflammatory cytokine TNF-α were significantly up regulated (p < 0.05, n = 5 per group) in the retina at 1–4 weeks after laser injury and PBS treatment. However, the TGF-β inhibitor LY2157299 could significantly inhibit VEGF and TNF-α protein expressions in the retina at 2–4 weeks when compared with that of PBS controls during the development of Laser-induced CNV formation (p < 0.05, n = 5 per group).

In addition, the levels of phosphorylated Smad2/3 (P-Smad2/3, Fig. 3D–F) increased significantly (p < 0.05, p = 5 per group) in the retina at 2–3 weeks after Laser injury and PBS treatment when compared with that of normal control. Similarly, the increase of P-Smad2/3 was significantly suppressed (p < 0.05, n = 5 per group) in the retina at 2–3 weeks after Laser injury and LY2157299 treatment. These results suggest that TGF-β/Smad signaling pathway may be involved in the high expressions of VEGF and TNF-α in retina following Laser-induced CNV formation.

### Effect of Decorin on VEGF and TNF-α Protein Expression and Smad2/3 Phosphorylation Levels in Retina Following Laser-induced CNV Formation

**Figure 4:**
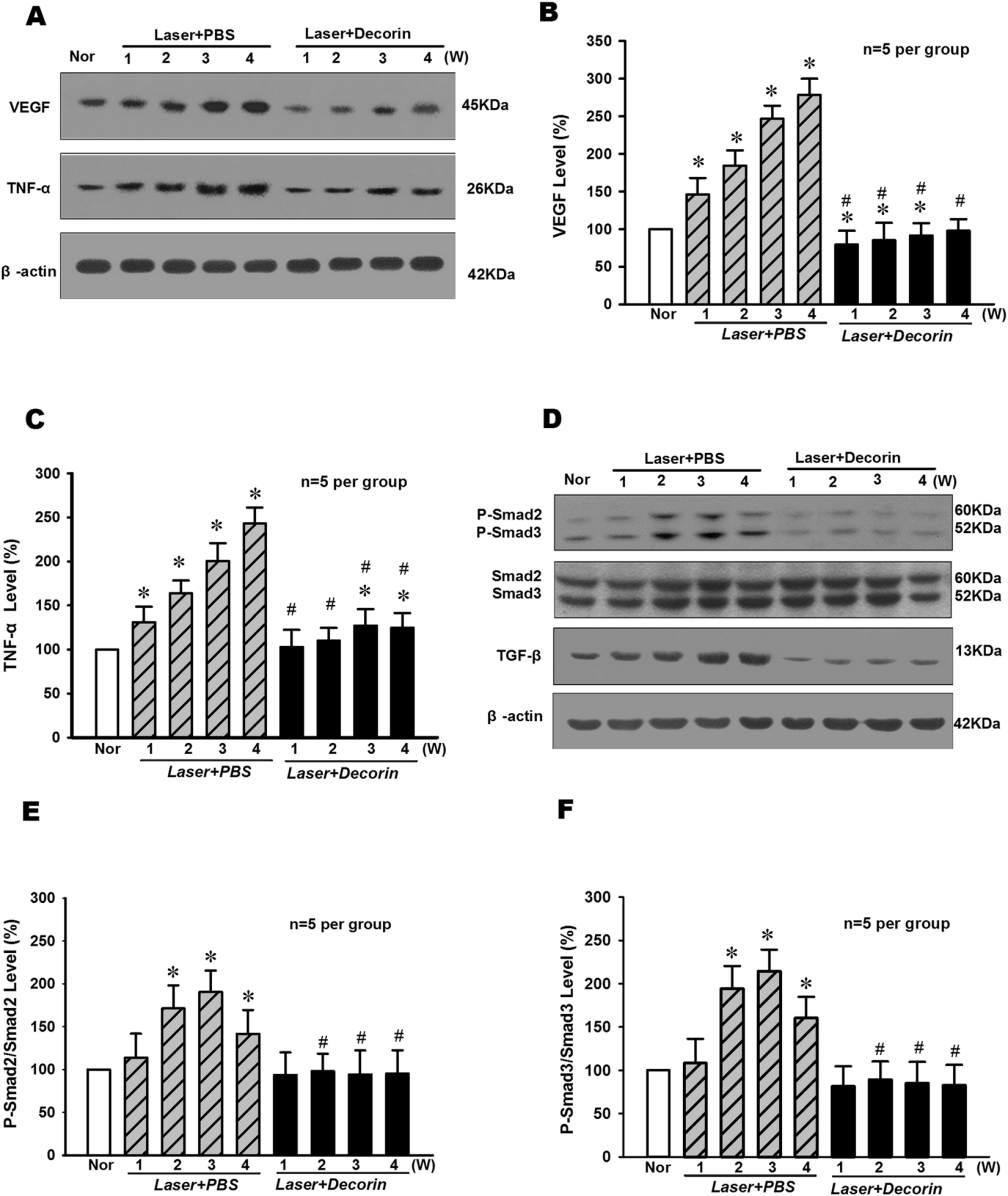
Effect of TGF-β natural inhibitor Decorin on the levels of VEGF and TNF-α protein expression and Smad2/3 phosphorylation in RPE-choroid complex of mice after Laser-induced CNV formation. (**A**)The typical result of Western blot showed changes of VEGF and TNF-α protein expressions. The quantitative analysis results demonstrated that the protein expression levels of VEGF (**B**) and TNF-α (**C**) were significantly elevated following CNV induction, but TGF-β inhibitor Decorin (intravitreal injection, 1 μl) treatment could significantly suppress the increase of VEGF and TNF-α protein expressions in RPE-choroid complex of mice at 1–4 weeks after Laser-induced CNV formation. (**D**) The representative result of Western blot showed the effect of Decorin on Smad2/3 phosphorylation and TGF-β protein expression levels in RPE-choroid complex of Laser-treated mice. The quantitative analysis results indicated that Decorin could significantly inhibit the increase of p-Smad2/Smad2 (**E**) and p-Smad3/Smad3 (**F**) levels in RPE-choroid complex of mice after Laser-induced CNV formation. The TGF-β protein levels were significantly suppressed in RPE-choroid complex of mice after Decorin treatments. *P < 0.05 vs Nor; ^#^P < 0.05 vs corresponding PBS treatment; n = 5 per group.

TGF-β natural inhibitor Decorin has the same effect with LY2157299 in blocking the laser-induced expression of VEGF, TNF-α and Smad2/3. As shown in Fig. 4A–C, Decorin treatment could significantly suppress the increase of VEGF and TNF-α protein expressions in RPE-choroid complex of mice at 1–4 weeks after Laser-induced CNV formation (p < 0.05, n = 5 per group). And besides, Fig. 4D–F indicated that Decorin could significantly inhibit the increase of P-Smad2/3 levels in RPE-choroid complex of mice after Laser-induced CNV formation (p < 0.05, n = 5 per group). The TGF-β protein levels were significantly suppressed in RPE-choroid complex of mice after Decorin treatments (p < 0.05, n = 5 per group).

### Effects of LY2157299 and Decorin on Laser-induced CNV Formation in Retina of Mice

**Figure 5:**
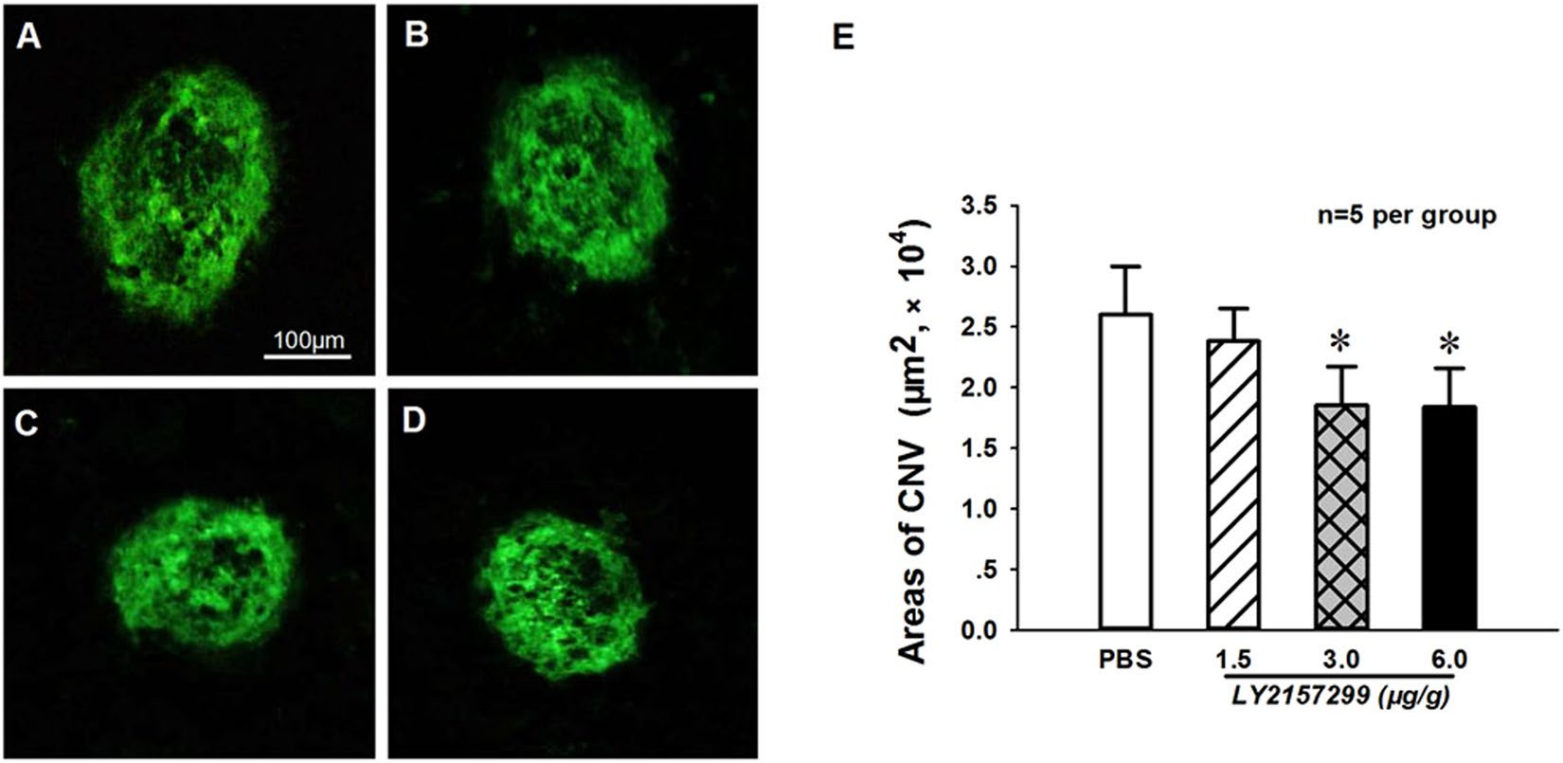
TGF-β inhibitor LY2157299 could dose-dependently inhibit the Laser-induced CNV formation at in RPE-choroid-sclera complex of mice. The representative images showed CNV staining with isolectin-B4 in the RPE-choroid-sclera complex of Laser-treated mice at 2 weeks after intraperitoneal injections of PBS (**A**) and LY2157299 at 1.5 (**B**), 3.0 (**C**) and 6.0 μg/g BW (**D**). (**E**) The statistical results suggest that LY2157299 (3.0–6.0 μg/g BW) could significantly inhibit Laser-induced neovascular growth when compared with that of PBS treatment. *P < 0.05 vs PBS treatment; bars = 100 μm; n = 5 per group.

To further determine the involvement of TGF-β/Smad in retinal CNV formation through regulation of the inflammatory and angiogenic factors that promote neovascularization, the effects of TGF-β chemical inhibitor LY2157299 and natural inhibitor Decorin on Laser-induced CNV formation were observed in the retina of mice. The C57BL/6 J mice right after Laser injury were intraperitoneally injected either with 1.5, 3.0, 6.0 μg/g BW of LY257299 or PBS containing 0.07% DMSO in the controls. As shown in Fig. 5A–E, the isolectin-B4 staining results of RPE/choroid flat mounts indicated that 3.0–6.0 μg/g BW of LY257299 could significantly (P < 0.05, n = 5 per group] reduce the neovascular area in retina of mice at day 14 after Laser-induced CNV formation when compared with that of PBS treated control groups.

**Figure 6:**
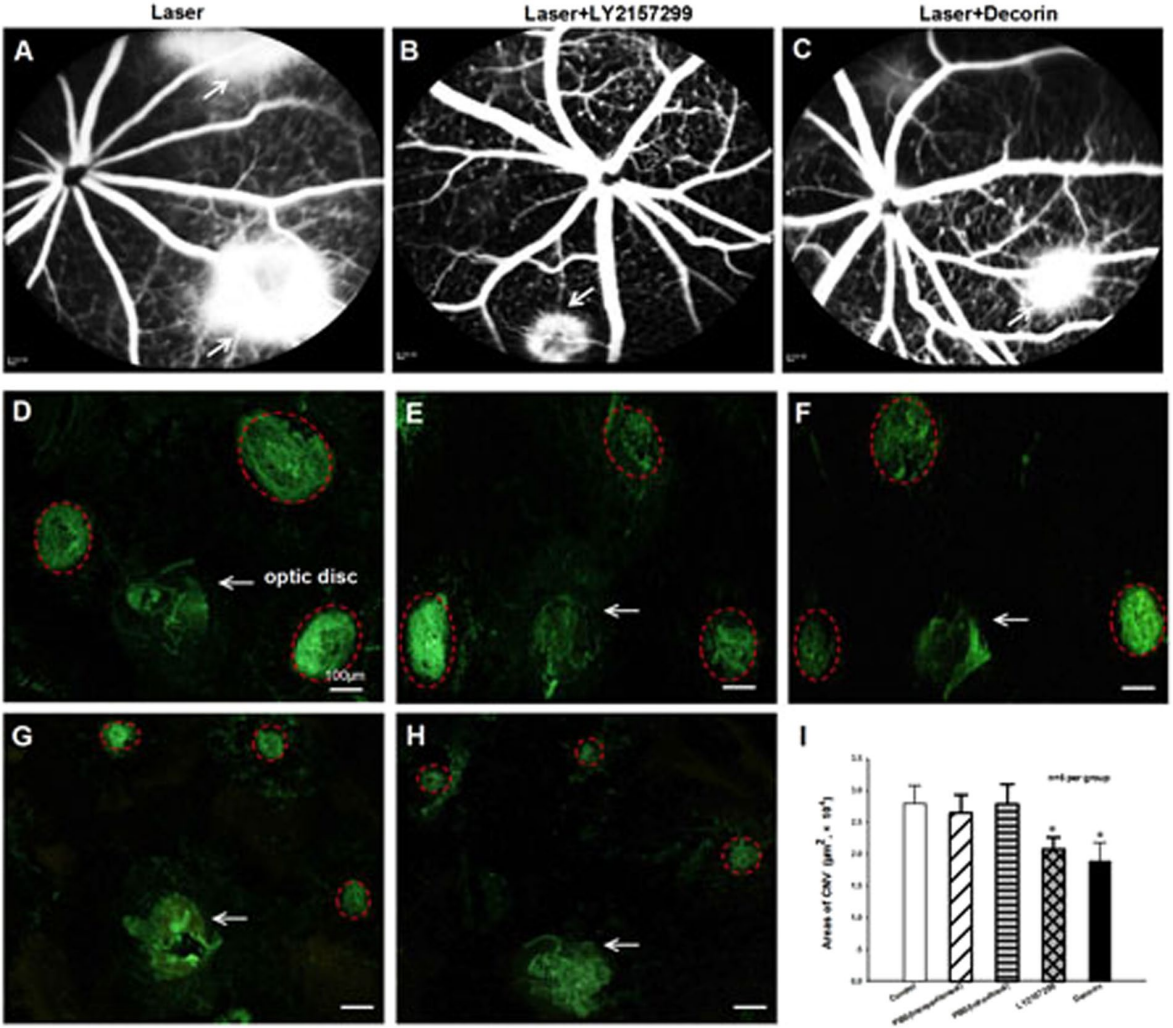
TGF-β inhibitors LY2157299 and Decorin could suppress the Laser-induced CNV formation in RPE-choroid-sclera complex of mice. The representative FFA images showed the fluorescence leakage in eye fundus of vehicle (**A**), TGF-β chemical inhibitor LY2157299 (intraperitoneal injection of 3.0 μg/g BW, **B**) and TGF-β natural inhibitor Decorin (intravitreal injection of 10 μg in 1 μl PBS, **C**) treated mice at 2 weeks after Laser photocoagulation (white arrowheads indicated Laser-injured spots). Fluorescein leakage was reduced and no abnormalities were observed in the retinal blood vessels of Laser-injured mice after LY2157299 and Decorin treatments. The isolectin-B4 stained images showed the changes of CNV area in RPE-choroid flat mounts of mice following Laser injury only (**D**), intraperitoneal injection of PBS (**E**) and LY2157299 (**G**), and intravitreal injection of PBS (**F**) and Decorin (**H**) at 2 weeks after photocoagulation. Red dotted lines indicate CNV lesions (bar = 100 µm). (**I**) The areas of CNV lesions were quantified by using digital imaging analysis, and showed that the CNV size was significantly suppressed by both LY2157299 and Decorin treatments when compared with that of controls. *P < 0.05 vs corresponding PBS treatment; n = 5 per group.

In addition, the effect of a naturally occurring TGF-β inhibitor Decorin (10 μg in 1 μl PBS, intravitreal injection) on the development of CNV after Laser photocoagulation was determined by using fluorescence leakage and isolectin-B4 staining areas. The representative FFA images showed that both LY257299 (3.0 μg/g BW intraperitoneal injection, Fig. 6B) and Decorin (10 μg intravitreal injection, Fig. 6C) could reduce fluorescein leakage when compared to the eyes treated with PBS (Fig. 6A) at day 14 after Laser photocoagulation. As shown in Fig. 6D–I, the quantitative analysis results of RPE/choroid flat mounts stained with isolectin-B4 also demonstrated significant reduction (P < 0.05, n = 5 per group) in CNV formation areas in retina for both LY2157299 and Decorin treatments when compared with that of their corresponding PBS control mice at day 14 after Laser-induced CNV formation.

### Regression of Established CNV after Decorin Treatment

**Figure 7:**
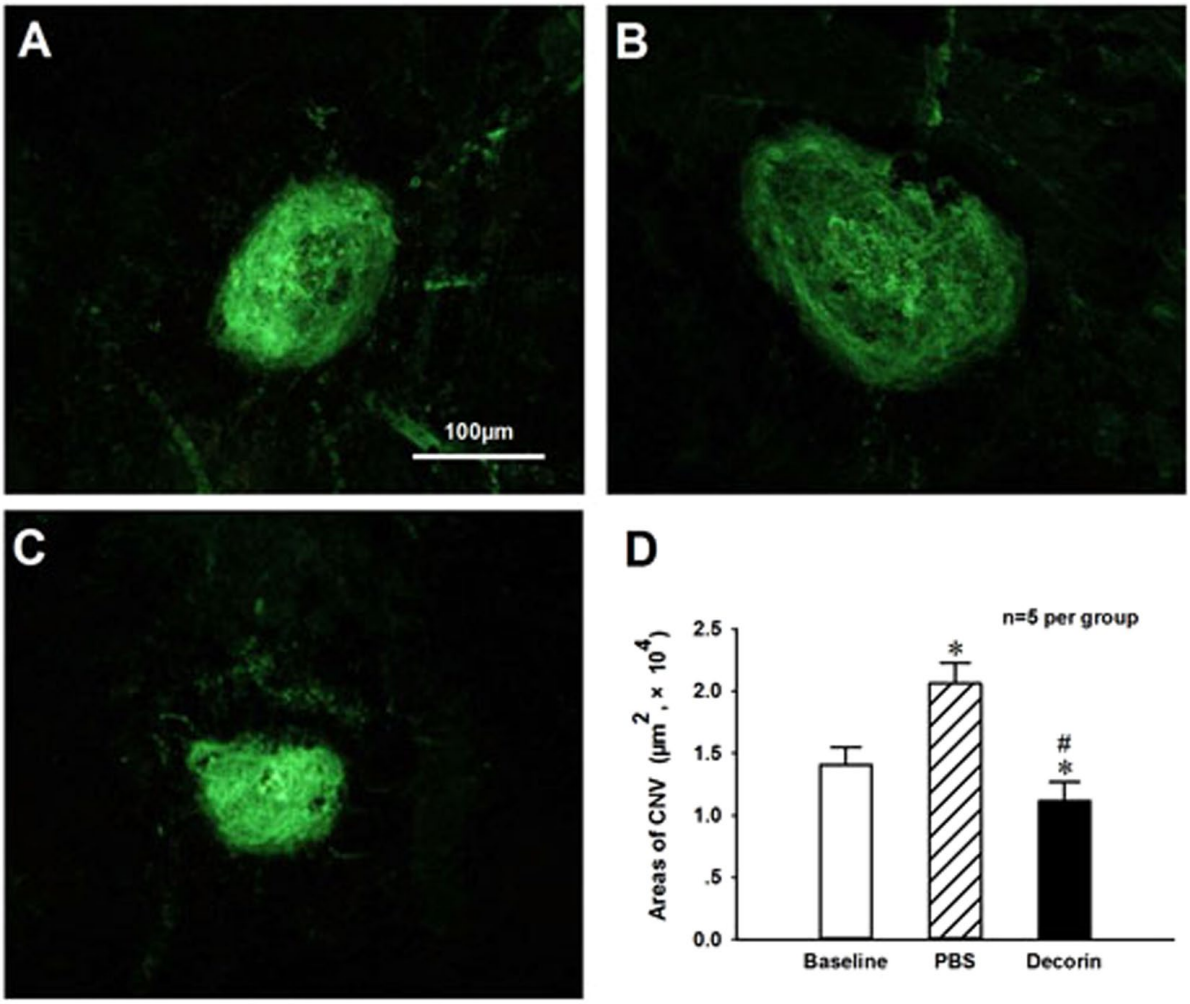
Regression of established CNV formation in RPE-choroid-sclera complex of mice after intravitreal injection of TGF-β inhibitor Decorin. Decorin or PBS was delivered by intravitreal injection at one week after Laser-induced CNV formation. The representative results of isolectin-B4 staining in choroid flat mounts were obtained at one week as baseline (**A**), or two weeks after one-week PBS (**B**) and Decorin (10 μg in 1 μl PBS, **C**) treatments following Laser-induced CNV formation. The digital imaging analysis results demonstrated that Decorin could significantly improve the regression of established CNV formation (**D**). *P < 0.05 vs baseline; ^#^P < 0.05 vs PBS treatment; n = 5 per group.

To test if TGF-β inhibitor attenuate CNV development after neovascularization has started, the CNV area was measured in a cohort of mice at day 7 after Laser-photocoagulation to provide the baseline (Fig. 7A). The intravitreal injections of Decorin (10 μg in 1 μl PBS) or PBS were initiated at day 7 after Laser-photocoagulation, and then additional week later, the CNV areas on RPE/choroid flat mounts stained with isolectin B4 were analyzed (Fig. 7B and C). The quantitative analysis results (Fig. 7D) revealed that the mean CNV area in eyes treated with Decorin was significantly less than those seen in Baseline and PBS treated control eyes (P < 0.05, n = 5 per group), suggesting a regressive effect of Decorin on established CNV. These data demonstrated that TGF-β inhibitor Decorin could inhibit Laser-induced CNV formation whether administered prior to or after the onset of angiogenesis.

## Discussion

Wet AMD with the hallmark presence of CNV is one of the main causes of blindness in the world. Understanding the mechanisms of CNV etiopathology could have a better impact on the health and quality of life of patients with wet AMD. Intravitreal injection of anti-VEGF antibody is currently considered the gold standard therapy for CNV, albeit with over 60% of AMD patients do not have improved vision after the treatment^29^. In addition, some commonly used treatments, such as Laser photocoagulation, photodynamic therapy or intravitreal administration of triamcinolone, also have limited efficacy and severe side effects^30, 31^, other alternative therapeutic strategies are being sought.

As Laser-induced CNV by a rupture of Bruch’s membrane shares many of the same pathologic steps with neovascularization arises from varied causes, we utilized this *in vivo* model to evaluate the effect of TGF-β signaling pathway because it has been shown to possess some actions including inflammatory and angiogenesis. Laser lesions showed the progressive subretinal ingrowth of new vessels from the early onset at one week up to peak at four weeks after photocoagulation. Using the mouse model of CNV, we demonstrated the relationship between TGF-β signaling and CNV. We observed that local expression of TGF-β in the retina-choroid complex submitted to Laser-induced CNV is upregulated during experimental CNV development, but there was a low expression level in the normal mice. Administration of the TGF-β inhibitors prior to or after the onset of angiogenesis potently attenuated CNV lesion size, which was associated with suppressing the levels of Smad2/3 phosphorylation and the expressions of VEGF and TNF-α in the injured eye. Our results suggest that TGF-β/Smad signaling pathway inhibition have potential implications for the novel therapeutic of wet AMD, different to that of current treatments for CNV lesions.

Neovascularization is regulated by complex interactions among numerous cytokines and growth factors in a highly orchestrated manner, which has become a major area of research in recent years. Although the pathogenesis underlying CNV has not been fully elucidated, increasing experimental and clinical studies have revealed that this multifactorial process involves multiple molecules, including inflammatory cells and cytokine networks. These elements may come from the serum as a consequence of blood-ocular barrier breakdown, or from infiltrating leucocytes and resident cells in diseased ocular tissue^32^. The CNV tissues from human surgical extracted samples and the rodent Laser-induced models express high levels of proangiogenic molecules and decreased production of angiogenic inhibitors. Studies confirm that VEGF is the central link in promoting angiogenesis in the development of CNV and other retinal neovascularization disorders^17^. However, there remain some questions as to why VEGF inhibitions are only partially effective. Apart from VEGF, many other cytokines and growth factors contribute to CNV either directly via activation of their cognate receptors or indirectly via crosstalk with VEGF and other signaling pathways. More recently, studies have been directed towards other mediators, among which is TGF-β. TGF-β is a multifunctional regulator in the retina that participates in pathological angiogenesis and neovascularization. It regulates expression of a lot of genes in epithelial cells (ECs), particularly those involved in establishment of, and interaction with, the basement membrane (BM)^33^. Unfortunately, the mechanisms of TGF-β in the formation of angiogenesis *in vivo* have not been well defined.

The TGF-β mRNAs expression during the process of neovascularization could be detected in retinal pigment ECs, fibroblast-like cells and the endothelium of the neovascular region^32^. We report here that TGF-β protein expression is up regulated during Laser-induced CNV development. TGF-β has a crucial role in the formation of extracellular matrix. It executes the production of matrix molecules and represses matrix degradation by stimulation of protease inhibitor synthesis and inhibition of proteases synthesis^34^. The up-regulation of TGF-β strongly suggested that it could be involved in the accumulation of extracellular deposits such as drusen. Previous reports have revealed that TGF-β strongly enhanced VEGF mRNA expression and protein secretion, and synergistically associated with TNF-α over VEGF transcription^32, 35, 36^. Some studies also confirmed that TNF-α is a pleiotropic cytokine which mediates angiogenic and inflammatory effects in the cells involved in the formation of CNV^37^. Furthermore, TGF-β activation may contribute to the inflammatory cell-induced neovascularization since it is highly potent chemotactic agents for monocytes. These findings led us to speculate that TGF-β may work in concert with other cytokines in VEGF up-regulation, and surmise that selective blockage of TGF-β would prevent the formation of CNV. In this study, we found that the Laser injury-induced increase of TNF-α and VEGF protein expressions could be suppressed by TGF-β blockage during CNV progression, and the TGF-β chemical inhibitor LY2157299 and natural inhibitor Decorin could prevent CNV formation and improve its regression, suggesting that TGF-β is a critical trophic factor for the expression of aberrant neovascularization.

In addition, we further explored the possible pathways through which TGF-β promotes neovascularization. Studies provide evidences that TGF-β signal transduction includes both specific Smad-dependent and Smad-independent pathways that can be activated by the TGF-β receptors via either phosphorylation or direct interaction^32^. Briefly, the canonical intracellular TGF-β/Smad signaling initiates its regulatory functions binding to the TβRII located in the cell membrane, the constitutively active TβRII activates TβRI, which phosphorylates downstream regulatory Smad2/3. Then the phosphorylated Smad2/3 oligomerize with the Smad4 and form the Smad complex, which translocate into the nucleus in combination with transcription factors^38^. A series of previous reports have indicated that TGF-β signaling promotes tumor formation and angiogenesis^39^. But on the contrary to the above findings, TGF-β has been shown to play a tumor suppressing role through many different mechanisms including inhibition of cell proliferation and induction of apoptosis^40^. To these studies, it has shown that TGF-β plays a dual role in tumor. Such complexity regulation of this differential signaling is contingent on the concentration of TGF-β, the presence or absence of other regulatory factors and the cells types^41^. Our current results showed that TGF-β/Smad pathway may function as an angiogenesis promoter in CNV development, and the injection of TGF-β type I receptor inhibitor LY2157299 [a new type I receptor TGF-β kinase antagonist^27^] resulted in significantly inhibition of phosphorylated Smad 2/3. However, this treatment did not further change TGF-β expression. We noticed that there is a continuous increasing of TGF-β, VEGF and TNF-α with the peak at 3–4 weeks post-laser, the same time interval required for the onset of neovascularization. The suppression of activated Smad 2/3 is consistent with the results of the significant decrease in protein expressions of pro-angiogenic molecules including VEGF and TNF-α. This supports the hypothesis that LY2157299 suppresses CNV development via inhibition of the TGF-β/Smad pathway, and the possible effects of TGF-β on neovascularization may involve inducing the accumulation of TNF-α, in addition to stimulating VEGF overexpression by the RPE. In fact, Smad pathway is integrated into a signaling cross-talk network, involved in a variety of other signaling pathways. Much remains to be elucidated regarding this mechanism.

In conclusion, the results firstly demonstrated that TGF-β/Smad signaling plays an important role in angiogenesis of Laser-induced CNV formation of wet AMD. TGF-β inhibition by its specific inhibitors LY2157299 and Decorin is able to reduce CNV progression through the Smad signaling pathway, which was achieved through down-regulation of VEGF and TNF-α. These results suggest that TGF-β inhibitors have great potential in wet AMD treatment, providing an alternative to traditional methods in the clinical field and merits further investigation by *in vivo* studies.
